# Association of qEEG TAR and TBR During Eyes-Open and Eyes-Closed with Plasma Oligomeric Amyloid-β Levels in an Aging Population

**DOI:** 10.3390/jcm14228069

**Published:** 2025-11-14

**Authors:** Chanda Simfukwe, Seong Soo A. An, Young Chul Youn, Jeena Kang

**Affiliations:** 1 Department of Bionano Technology, Gachon University, 1342 Seongnam-daero, Seongnam-si 13120, Republic of Korea; chandaelizabeth94@gmail.com; 2 Department of Neurology, College of Medicine, Chung-Ang University, Seoul 06974, Republic of Korea; 3 Department of Medical Informatics, Chung-Ang University College of Medicine, Seoul 06974, Republic of Korea; 4 Department of Animal Science and Technology, Chung-Ang University, Seoul 06974, Republic of Korea; jeena0138@cau.ac.kr

**Keywords:** Alzheimer’s disease, cognitive impairment, aging, amyloid oligomers, power spectrum density, electroencephalography

## Abstract

**Background/Objective**: Timely and successful treatments for Alzheimer’s disease (AD) depend on early detection. The Multimer Detection System (MDS-OAβ) for quantifying plasma oligomeric amyloid-β (OAβ) has shown promise as a biomarker of amyloid disease. The theta-to-alpha ratio (TAR) and theta-to-beta ratio (TBR) are two examples of spectral power metrics that can be used in resting-state quantitative EEG (qEEG) to evaluate brain function non-invasively. This study used resting-state EEG (rEEG) recordings obtained while the subjects were both eyes-open (EO) and eyes-closed (EC) to investigate the relationship between regional qEEG power ratios and plasma MDS-OAβ levels in older adults. **Methods**: The analysis comprised 174 patients between the ages of 60 and 85, with 2 in the low-MDS-OAβ group and 82 in the high-MDS-OAβ group. The clinical plasma cutoff was 0.78 ng/mL. All participants underwent rEEG recordings and plasma OAβ quantification. EEG pre-processing included bandpass filtering (0.5–100 Hz), average re-referencing, artifact rejection using independent component analysis (ICA), and spectral power estimation using Welch’s method. The TAR and TBR were calculated across five lobar regions (frontal, central, parietal, occipital, and temporal) during both EO and EC conditions. To normalize data distributions, EEG ratio variables were log-transformed prior to statistical analysis. Group comparisons and linear regression analyses were conducted to evaluate the associations between EEG power ratios and MDS-OAβ levels. Adjusted regression models included age, years of education, and neuropsychological test scores as covariates. Statistical significance was set at *p* < 0.05. **Results**: No significant associations were found between TAR and plasma MDS-OAβ levels across any lobar regions under either EO or EC conditions. In contrast, TBR exhibited consistent and significant negative associations with MDS-OAβ levels, particularly under EC conditions. Adjusted regression models revealed that higher MDS-OAβ levels were associated with lower TBR values in the central (β = −0.059, *p* = 0.015), parietal (β = −0.072, *p* = 0.006), occipital (β = −0.067, *p* = 0.040), and temporal (β = −0.053, *p* = 0.018) lobes, with the strongest inverse relationship observed in the parietal lobe. A similar, though slightly weaker, pattern was observed during EO conditions, with significant inverse associations in the frontal, central, and temporal lobes. **Conclusions:** Our findings indicate that, after adjusting for covariates, increased plasma MDS-OAβ levels are significantly associated with a reduced TBR, particularly in the parietal and central lobes, under both EO and EC resting-state conditions. In contrast, no significant associations were observed with TAR. These results suggest that a lower TBR may reflect an increased peripheral amyloid burden and highlight its potential as a sensitive qEEG biomarker for early amyloid-related brain changes in older adults.

## 1. Introduction

Alzheimer’s disease (AD) is a chronic, progressive neurodegenerative disorder marked by a gradual decline in cognitive abilities, ultimately impairing an individual’s capacity to perform everyday tasks independently [1]. The development of efficient diagnostic biomarkers and treatment approaches depends on the early recognition of neurodegenerative alterations [2]. The aggregation of β-amyloid (Aβ) is regarded as a hallmark of AD and functions as a crucial biomarker for diagnosis and prognosis [1,3]. The aggregation of Aβ is linked with synaptic failure and neuronal death, especially in areas of the cortex and hippocampal regions that are important for memory and cognition [3,4]. The β- and γ-secretase enzymes sequentially cleave amyloid precursor protein (APP) to produce Aβ. This process starts with the formation of monomeric species, which then gradually unite into oligomers, fibrils, and finally amyloid plaques [3,4,5]. Oligomeric Aβ (OAβ), which is thought to be the most neurotoxic of these forms, has been closely associated with synaptic disruption and the advancement of AD [6,7].

Biomarkers are essential for the diagnosis, staging, and risk stratification of AD [8,9]. In clinical trials for disease-modifying medicines, biomarkers are crucial for identifying appropriate participants, forecasting treatment response, and evaluating therapeutic efficacy [8,9,10]. Currently, approved amyloid biomarkers encompass magnetic resonance imaging (MRI), functional MRI (fMRI), positron emission tomography (PET), and cerebrospinal fluid Aβ_1–42_ concentrations [11,12,13]. These procedures are useful, but they have several problems: they are invasive and expensive, and results can vary from lab to lab [14]. Consequently, considerable focus has shifted to the advancement of blood-based biomarkers that provide a less invasive and more scalable option [15].

Blood-based assays have recently been introduced to detect oligomeric Aβ, with improvements in accessibility and cost-effectiveness compared to CSF or imaging-based approaches. Among these, the Multimer Detection System–Oligomeric Aβ (MDS-OAβ) has shown considerable promise for AD detection. This technique measures the oligomerization tendency of spiked synthetic Aβ in plasma samples, based on a competitive sandwich immunoassay platform that utilizes capture and epitope-overlapping detection antibodies. These antibodies preferentially bind to oligomeric and multimeric Aβ species over monomers [16]. This approach, initially developed for protein misfolding disorders, has been adapted to quantify Aβ-related changes in peripheral blood [16,17].

The MDS-OAβ detects dynamic changes in circulating Aβ seen in AD and provides a new way to look for early signs of amyloid disease. An increasing number of studies show that plasma MDS-OAβ levels are highly linked to both the prevalence and degree of cognitive impairment. These links are stronger than those seen for standard Aβ plaque load [18,19]. Prior research has shown that MDS-OAβ levels are highly associated with the recognized clinical indicators of AD and display a high diagnostic efficacy, with a sensitivity of 78.3% and a specificity of 86.5% in differentiating AD patients from healthy controls [20]. Moreover, another study indicated a strong diagnostic accuracy for MDS-OAβ, with area under the curve (AUC) values between 0.84 and 0.87 [19,21,22]. Moreover, MDS-OAβ levels have demonstrated the capacity to predict aberrant amyloid PET scan outcomes, with an AUC of 0.77, which increased to 0.86 when integrated with age and apolipoprotein E epsilon 4 (APOE ε4) status [23]. Taken together, these findings support the utility of plasma MDS-OAβ as a reliable biomarker of cerebral amyloid pathology.

Electroencephalography (EEG) has currently been used as a valuable biomarker for the early diagnosis and detection of AD and other forms of dementia through quantitative EEG (qEEG) analysis [24,25,26]. Resting-state EEG (rEEG) data are commonly acquired during eyes-open (EO) and eyes-closed (EC) conditions, in which participants remain awake but refrain from engaging in any specific tasks or physical movements [27]. rEEG studies have consistently shown that AD is associated with a slowing of cortical rhythms, characterized by a reduced power in higher frequency bands, namely alpha (8–13 Hz) and beta (13–30 Hz), and an increased power in lower frequency bands, including delta (0.5–4 Hz) and theta (4–8 Hz) [28,29]. Similar, though less pronounced, spectral changes have been observed in individuals with mild cognitive impairment (MCI), a transitional state between normal aging and dementia [30].

In dementia studies, power spectral density (PSD) in qEEG analysis remains a fundamental and widely used technique for extracting physiologically meaningful information from brain neural activity [14]. Unlike traditional EEG interpretation, qEEG offers an objective and data-driven approach to characterizing large-scale brain dynamics by quantifying frequency-specific power across cortical regions [31,32]. PSD specifically reflects the aggregate oscillatory activity of cortical neurons and serves as a robust indicator of underlying neural function and organization [14,25]. Among the available spectral estimation methods, Welch’s method is most used due to its ability to reduce spectral variance by averaging modified spectral estimates across overlapping segments, thereby balancing frequency resolution and noise suppression more effectively than traditional Fast Fourier Transform (FFT) techniques [33,34,35]. The derived frequency power ratios of PSD-based qEEG analysis, particularly the theta-to-alpha ratio (TAR) and theta-to-beta ratio (TBR), have garnered considerable attention as promising biomarkers for AD [35].

These ratios serve to capture the relative balance between slow (theta) and fast (alpha or beta) neural oscillations, which are often disrupted in neurodegenerative conditions [35,36]. Recent research evidence suggests that TAR and TBR are sensitive to early AD-related changes in brain activity, with several studies reporting consistent alterations in these indices among individuals with AD compared to those who are cognitively healthy [37,38]. TBR has shown a high diagnostic accuracy in early-onset AD, supporting its potential clinical utility for early detection [37]. Both TAR and TBR demonstrate significant group-level differences and a clear separability across diagnostic categories, particularly when computed over extended time windows, which enhances the stability and reliability of these metrics [35,36,37,38]. These findings underscore the relevance of TAR and TBR as non-invasive, electrophysiological markers that reflect disease-specific neural dysfunction and hold promises for improving the early diagnosis and staging of AD.

Cognitive decline in AD is strongly driven by progressive synaptic dysfunction and loss [39,40,41]. Accumulating evidence from both clinical and preclinical studies suggests that Aβ plaque deposition contributes to localized synapse degeneration and impairs neurophysiological processes, such as memory formation and synaptic plasticity [42,43]. EEG, as a sensitive tool for detecting large-scale neural dynamics, has been increasingly utilized to capture the functional disruptions associated with AD pathology, particularly at the synaptic level through qEEG [25]. However, the extent to which rEEG-derived power ratios, specifically the TAR and TBR, reflect peripheral Aβ burden remains unclear. We hypothesized that elevations in TAR and TBR, indicative of a shift toward slower oscillatory activity, may be associated with a greater amyloid pathology. To investigate this, we analyzed rEEG data recorded during both EO and EC conditions, examining their associations with plasma oligomeric Aβ levels measured using MDS-OAβ in the adult population.

## 2. Materials and Methods

### 2.1. Demographics of the Participants

This study enrolled older adults between May 2015 and December 2017 as part of the nationally funded “*Dementia Overcoming Project in Korea*,” conducted at Chung-Ang University Hospital (Seoul, South Korea), which aims to develop protein biomarkers and advance early dementia research. A total of 178 individuals aged 60 to 85 years (mean age = 74.87 ± 8.52 years) were consecutively enrolled from the outpatient neurology clinic of Chung-Ang University Hospital. All participants were community-dwelling older adults who voluntarily took part following routine dementia screening. Although they reported subjective cognitive complaints, they remained functionally independent. Therefore, while recruitment occurred at a single site, the cohort reflects typical aging adults undergoing clinical evaluation for cognitive health. This study followed a cross-sectional observational design consistent with established reporting standards for observational research. Data collection and analysis were conducted by investigators blinded to participant identifiers to minimize bias.

All participants underwent rEEG, plasma MDS-OAβ analysis, and baseline assessments, including demographic data (age, sex, and years of education), as well as standardized neuropsychological evaluations. The study protocol was approved by the Institutional Review Board and Ethics Committee of Chung-Ang University Hospital (IRB Approval No. 2009-005-19331), and written informed consent was obtained from all participants prior to enrollment, following the standards of the Declaration of Helsinki.

Inclusion criteria required participants to report subjective cognitive decline and demonstrate the ability to complete clinical evaluations independently. Exclusion criteria comprised a diagnosis of major neurological disorders: brain infarction, cerebral hemorrhage, Parkinson’s disease, or other severe medical or psychiatric conditions. Participants receiving medications known to alter EEG activity (e.g., sedatives, antiepileptics, or psychotropics) were also excluded. Additionally, neurologists evaluated participants for cerebrovascular risk factors, depressive symptoms, and sleep disturbances; individuals with clinically significant abnormalities in any of these domains were excluded from enrollment. Trained neurologists screened all candidates to determine their eligibility, and medical experts conducted diagnostic interviews before final classification. Each participant completed a Korean-based neuropsychological test battery and routine laboratory assessments, including assessments for cholesterol, thyroid function, and vitamin B12 levels. Common age-related comorbidities such as hypertension and diabetes were allowed if clinically stable and comparable between groups.

### 2.2. Assessment of Amyloid-β Oligomerization in Plasma

The AlzOn™ test (PeopleBio Inc., Seongnam-si, Gyeonggi-do, Republic of Korea) was used to quantify plasma levels of OAβ, as described by Youn et al. [19]. This test uses the MDS, a modified enzyme-linked immunosorbent assay (ELISA) specifically designed to detect oligomeric forms of Aβ. The MDS method uses monoclonal antibodies that overlap epitopes and selectively target the N-terminal region of Aβ. This makes it easier for the antibodies to bind to oligomers instead of monomers [43]. This work entailed the overnight coating of 96-well black plates at 4 °C with monoclonal antibody 6E10 (BioLegend, San Diego, CA, USA) at a concentration of 3 (microgram per milliliter) μg/mL in carbonate–bicarbonate buffer (Sigma-Aldrich, St. Louis, MO, USA). The plates were then left at room temperature for two hours with 0.4% Block Ace and then washed with phosphate-buffered saline (PBS). The plates that had been prepared were kept at 4 °C until they were needed.

Plasma samples were frozen at 37 °C for 15 min before the experiment. Each reaction mixture had 10 μL of plasma, 4 μL of a heterophilic blocking reagent-1 (HBR-1), a HAMA blocker (Scantibodies Laboratory, Santee, CA, USA), and PBS-T. Synthetic Aβ was then added to this mixture, followed by a 1 h incubation at 37 °C to enhance oligomerization. Subsequently, 100 μL of the plasma mixture or standard dilutions were added to each well and incubated for 1 h at room temperature. Following three washes with Tris-buffered saline with Tween (TBST), the detection antibody WO2-HRP (Absolute Antibody Ltd., Lazenby, UK) diluted in TBST with 0.4% Block Ace was applied and incubated for an additional hour. Detection was carried out by adding 100 μL of 3,3′,5,5′-tetramethylbenzidine (TMB; Sigma-Aldrich, USA), and the enzymatic reaction was halted with 50 μL of 1 M sulfuric acid (H_2_SO_4_). Optical density (OD) values were then measured at 450 nm using a Victor 3 multi-spectrophotometer (PerkinElmer, Waltham, MA, USA). Plasma samples were processed and analyzed at a single certified laboratory (PeopleBio Inc., Republic of Korea) using identical reagent batches and standardized handling protocols. Consistent sample storage and assay procedures were maintained in accordance with the manufacturer’s guidelines to ensure measurement reliability and minimize inter-assay variability [19].

### 2.3. EEG Signals Acquisition and Pre-Processing

The EEG recordings were acquired utilizing the Comet AS40 amplifier system (GRASS; Telefactor, West Warwick, RI, USA) with gold-cup electrodes. The earlobes served as reference electrodes, and the electrode placement followed the international 10–20 system. There were 19 recording sites: Fp1, Fp2, F7, F3, Fz, F4, F8, T3, C3, Cz, C4, T4, T5, P3, Pz, P4, T6, O1, and O2 (see Supplementary Figure S1). Electrode-to-skin impedance was constantly checked and kept below 5 kΩ to guarantee high-quality data capture. Participants were seated comfortably in a quiet, controlled environment and instructed to remain relaxed yet alert throughout the recording to minimize variability in alertness, anxiety, and attention between the EO and EC conditions. The EEG waves were digitally captured and safely archived on a magnetic disk. Digital signal processing techniques, such as bandpass filtering, were used before data collection to separate pertinent brain oscillations while reducing noise and unnecessary frequencies. The frequency range of 0.5–100 Hz was chosen to minimize artifact contamination and record physiologically significant brain activity. Participants switched back and forth between EO and EC states during the recording session. Each condition had ten trials of 30 s, totaling about 5 min of raw data. EEG data were captured at 250 Hz. Following pre-processing and rigorous artifact rejection, at least 60 s of clean data per condition were retained for each participant to ensure the reliability of spectral analysis. Each participant contained an average of 45 artifact-free epochs, each lasting 4 s, totaling roughly 180 s for both the EO and EC conditions.

The raw rEEG data were processed using the EEGLAB toolbox (version 2024) in the MATLAB environment (R2024a; http://www.sccn.ucsd.edu/eeglab/ (accessed on 25 June 2025)). The pre-processing pipeline began with the first step, importing the raw EEG dataset, and the second step involved extracting epochs corresponding to the EO and EC conditions. The third step involved applying an average reference to minimize baseline variability across channels, leveraging the consistency of the 10–20 electrode layout and the high signal fidelity across participants. A bandpass filter (0.5–100 Hz) was then applied to attenuate slow drifts and high-frequency noise, preserving the physiologically relevant signal components. Artifacts were manually identified and excluded, after which independent component analysis (ICA) was performed using the RUNICA algorithm (https://sccn.ucsd.edu/~arno/eeglab/auto/runica.html accessed on 5 July 2025) to isolate and remove remaining ocular and muscular artifacts. Lastly, after artifact correction, PSD features were computed for canonical frequency bands (delta, theta, alpha, beta) across artifact-free epochs. The PSD for each epoch was estimated using Welch’s method with a 2 s Hamming window and 50% overlap. The resulting spectra were then normalized to total power within the 0.5–100 Hz range to compute the relative band power for each frequency band. These PSD measures served as the basis for subsequent statistical analyses. All pre-processing steps were performed using a standardized pipeline and identical parameters for every participant. Visual inspection after ICA ensured consistent artifact removal and comparable data quality across groups, minimizing potential bias due to ocular or muscle artifacts.

### 2.4. Neuropsychological Screening Batteries

Cognitive performance was assessed using the Seoul Neuropsychological Screening Battery (SNSB), one of the most used neuropsychological tests in South Korea, to determine if an individual has cognitive problems. The SNSB, given by qualified experts, tests a wide range of cognitive areas, such as language, memory, attention, executive functioning, and visuospatial processing [44,45,46,47]. All participants underwent a series of assessments, including the Korean Mini-Mental State Examination (K-MMSE), Rey Complex Figure Test (RCFT), Seoul Verbal Learning Test (recall), Digit Span Forward, Digit Span Backward, and the Korean Boston Naming Test.

### 2.5. Statistical Analysis

Using artifact-free EEG data, we found the regional TAR and TBR power ratios by dividing the relative theta band power by the alpha and beta band power, respectively. To obtain the mean lobar TAR and TBR indices for statistical analysis, we averaged the relative power spectral density (rPSD) values from all of the electrodes in each of the five cortical lobes: frontal (Fp1, Fp2, F3, F4, F7, F8, Fz), central (C3, C4, Cz), temporal (T3, T4, T5, T6), parietal (P3, P4, Pz), and occipital (O1, O2). Participants were categorized into low- and high-amyloid-load groups using a plasma MDS-OAβ cutoff of 0.78 ng/mL, as established by Youn et al. (2020) [19]. This cutoff was validated in a blinded clinical study, demonstrating 100% sensitivity and 92.3% specificity for distinguishing AD patients from cognitively normal controls. Adopting this clinically validated threshold ensured methodological consistency and comparability with previous research on plasma OAβ biomarkers. Assumptions of normality for parametric tests, TAR, and TBR values were subjected to log transformation. The Shapiro–Wilk test confirmed the normal distribution of the transformed data (*p* > 0.05).

Group comparisons of plasma MDS-OAβ concentrations and log-transformed EEG indices (TAR and TBR) across the five lobes were performed using independent two-sample t-tests. Pearson correlation coefficients were computed to assess linear associations between plasma MDS-OAβ levels and log-transformed EEG power ratios in each cortical region. Also, while adjusting for potential confounders, multiple linear regression analyses were conducted with log-transformed TAR and TBR as dependent variables and plasma MDS-OAβ as the independent variable. Covariates included age, years of education, and K-MMSE. Bonferroni correction was applied to adjust for multiple comparisons across lobes and EEG indices. All statistical analyses were performed using Python version 3.13.5 (https://www.python.org/ (accessed on 8 July 2025)) on the JupyterLab integrated development environment (IDE) (https://jupyter.org/ (accessed on 8 July 2025)), with a two-tailed significance threshold set at *p* ≤ 0.05.

## 3. Results

The study sample included 174 participants aged between 60 and 85 years (mean age = 74.87 ± 8.52 years), with the most common age group being 75–79 years (26.44%). Demographic and clinical characteristics are divided by MDS-OAβ level in Table 1. Individuals in the high-MDS-OAβ group (≥0.78 ng/mL) were, on average, older (76.07 ± 7.82 years) than those in the low-MDS-OAβ group (<0.78 ng/mL; 73.79 ± 9.01 years). The sex distribution was similar between the groups, with about 71% of the total sample being female. Educational levels varied slightly: 24.14% of all participants had less than a primary school education, while 33.33% had an education beyond college. In the high-MDS-OAβ group, 20.73% had not finished elementary school and 34.15% had post-secondary education, compared to 27.17% and 32.61%, respectively, in the low-MDS-OAβ group. The overall average years of education was 10.89 ± 4.44, with no significant difference between the two groups (low: 10.74 ± 4.49 years; high: 11.04 ± 4.41 years).

Cognitive function was assessed using a subset of tests from the SNSB. As shown in Table 2, there were no statistically significant differences in the mean test scores between the low- and high-MDS-OAβ groups across the three neuropsychological domains assessed. Specifically, the mean score for the K-MMSE was 23.75 (SD = 4.54) in the low-MDS-OAβ group and 23.02 (SD = 5.11) in the high group (*p* = 0.32). Similarly, performance on the Seoul Verbal Learning Test Recall was comparable between groups (low: 13.99 ± 5.83; high: 13.15 ± 5.14; *p* = 0.31). For the Digit Span Backward task, the low-MDS-OAβ group had a mean score of 3.86 (SD = 1.75) versus 3.50 (SD = 1.29) in the high group (*p* = 0.12). These findings indicate no significant differences in the general cognitive performance between participants classified as high- versus low-MDS-OAβ.

The brain lobe comparisons of EEG power ratios between the low- and high-MDS-OAβ groups are summarized in Table 3. Across the entire cohort, TBR was highest in the frontal lobe (mean = 0.76, SD = 0.26), followed by the parietal lobe (mean = 0.59, SD = 0.33). For TAR, the values were relatively low across all regions, with a negative mean observed in the occipital lobe (−0.15, SD = 0.33). When stratified by MDS-OAβ status, participants in the high-MDS-OAβ group exhibited slightly elevated mean values for both TAR and TBR across most lobes compared to the low-MDS-OAβ group. However, none of these differences reached statistical significance. For example, in the frontal lobe, the TBR was (mean ± sd) 0.75 (SD = 0.26) in the high-MDS-OAβ group versus 0.78 (SD = 0.25) in the low group (*p* = 0.40), while the parietal TBR was 0.56 (SD = 0.33) versus 0.61 (SD = 0.33), respectively (*p* = 0.30). Similarly, no significant differences were observed for TAR in the central (*p* = 0.85), occipital (*p* = 0.58), or temporal (*p* = 0.96) lobes. These results suggest that, although small variations in EEG band power ratios were present between groups, they were not statistically meaningful.

Table 4 summarizes the Pearson correlation coefficients between log-transformed EEG power ratios (TAR and TBR) and plasma MDS-OAβ levels across lobar regions under EO and EC conditions. For TAR, no statistically significant correlations were observed in either condition. In the central region, TAR showed negligible correlations (EO: r = −0.01, *p* = 0.39; EC: r = −0.02, *p* = 0.25), and, similarly, occipital TAR yielded nonsignificant associations (EO: r = 0.01, *p* = 0.72; EC: r = −0.01, *p* = 0.66). In contrast, central TBR showed a statistically significant negative correlation with plasma MDS-OAβ levels in both EO (r = −0.06, *p* = 0.02) and EC (r = −0.06, *p* = 0.01) states. Occipital TBR also exhibited weak negative correlations, though these did not reach statistical significance (EO: r = −0.06, *p* = 0.08; EC: r = −0.06, *p* = 0.07).

To investigate the relationship between plasma MDS-OAβ concentrations and EEG spectral indices during the EO and EC resting states, linear regression analyses were conducted for each lobar region with regression coefficients (β) and standard errors (SE). In Table 5a, showing the EO resting condition, both the unadjusted and adjusted models (including age, years of education, and K-MMSE) showed no statistically significant association between TAR and MDS-OAβ levels across any lobe. The regression coefficients for TAR were close to zero in all cases (unadjusted β range = −0.02 to 0.01; adjusted β range = −0.01 to 0.01), with *p*-values well above the significance threshold, indicating a negligible influence of plasma MDS-OAβ on theta–alpha dynamics under EO conditions. In contrast, the TBR demonstrated a consistent inverse relationship with MDS-OAβ across multiple regions. In the unadjusted model, higher MDS-OAβ levels were significantly associated with a lower TBR in the frontal (β = −0.04, *p* = 0.02), central (β = −0.06, *p* = 0.02), and temporal lobes (β = −0.06, *p* = 0.01), with a trend toward significance in the parietal lobe (β = −0.05, *p* = 0.05). The occipital lobe exhibited a negative but nonsignificant association (β = −0.06, *p* = 0.08). After adjusting, the inverse associations remained robust for the central (β = −0.05, *p* = 0.02), frontal (β = −0.04, *p* = 0.03), and temporal lobes (β = −0.05, *p* = 0.02). The parietal lobe demonstrated the strongest association in the adjusted model (β = −0.05, *p* = 0.06, borderline significance), while the occipital lobe retained a negative but nonsignificant trend (β = −0.05, *p* = 0.09).

Table 5b summarizes the linear regression models during the EC resting condition across lobar regions. For the TAR, neither the unadjusted nor adjusted models demonstrated significant associations in any region. Regression coefficients for TAR remained close to zero in all lobes (unadjusted β range = −0.03 to −8.00 × 10^−3^; adjusted β range = −0.03 to −7.00 × 10^−3^), with *p*-values consistently exceeding 0.1, indicating that MDS-OAβ exerted minimal influence on theta–alpha dynamics in the EC state. Contrary to the TAR, the TBR showed a robust pattern of inverse associations with MDS-OAβ. In the unadjusted models, higher MDS-OAβ levels were significantly associated with a lower TBR in the central (β = −0.06, *p* = 0.01), parietal (β = −0.07, *p* = 0.01), and temporal lobes (β = −0.05, *p* = 0.03), with the occipital lobe showing a negative but borderline association (β = −0.06, *p* = 0.07). The frontal lobe exhibited a nonsignificant trend (β = −0.03, *p* = 0.11). In the adjusted models, the strongest inverse association was observed in the parietal lobe (β = −0.07, *p* = 6.00 × 10^−3^), followed by significant effects in the central (β = −0.059, *p* = 0.02), occipital (β = −0.07, *p* = 0.04), and temporal lobes (β = −0.05, *p* = 0.02). The frontal lobe continued to show a weak, nonsignificant trend (β = −0.03, *p* = 0.12).

## 4. Discussion

**Figure 1:**
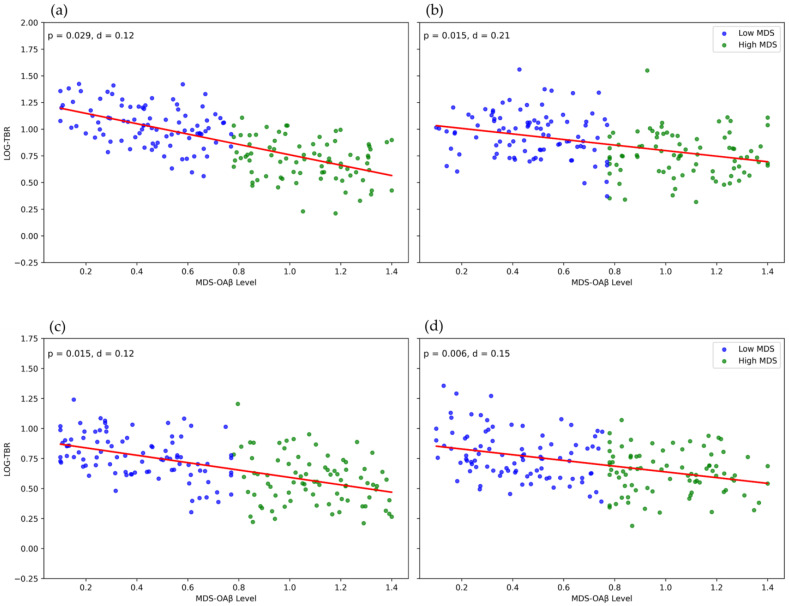
Linear regression plots of TBR association with plasma MDS-OAβ levels across lobar regions in EO and EC conditions. (**a**) Central lobe EO, (**b**) temporal lobe EO, (**c**) central lobe EC, (**d**) parietal lobe EC. Note: *p* = statistical significance, d = Cohen’s d (effect size, magnitude of group difference). Abbreviations: EO, eyes-open; EC, eyes-closed; LOG-TBR, logarithmic theta–beta ratio; MDS-OAβ: Multimer Detection System–Oligomeric Amyloid-βeta; *p*, probability value; d, Cohen’s d effect size.

Our investigation demonstrated a substantial correlation between MDS-OAβ and the log-transformed EEG power ratios, particularly TBR, in elderly individuals. Elevated MDS-OAβ levels were significantly correlated with a reduced TBR in the central and temporal lobes during EO, and in the central and parietal lobes during EC. In the EO condition, the frontal lobe exhibited a notable inverse correlation between MDS-OAβ and TBR, whereas, in the EC condition, this correlation was observed in the parietal and occipital lobes. These findings suggest that an increase in the amyloid burden may be linked to a decrease in the low-frequency band across these brain regions. No substantial correlations were detected for TAR across any of the lobes. The absence of significant TAR associations is unlikely to result from methodological factors, as both TAR and TBR were derived from identically pre-processed, log-transformed relative power values using the same segmentation and normalization pipeline. Instead, this null finding likely reflects genuine physiological specificity: TBR is more sensitive to the cholinergic and synaptic alterations associated with amyloid pathology, whereas TAR primarily reflects a general cortical slowing linked to attentional or arousal mechanisms [37,38], which may be less affected in this cohort. The linear regression plots (Figure 1) further illustrate these findings, showing strong, significant inverse relationships between MDS-OAβ and TBR during the EO (central and temporal lobes) and EC (central and parietal lobes) conditions.

AD is characterized by the deterioration of synaptic connections associated with the accumulation of neurotoxic Aβ in the brain, with synaptic dysfunction serving as a prominent signal of early cognitive impairment [48,49]. The amyloid cascade hypothesis posits that Aβ monomers assemble to create soluble Aβ oligomers [50]. Specific oligomers ultimately form amyloid plaques, while others cause neuronal damage, resulting in dementia [49,50]. The OAβ is the most neurotoxic variant of Aβ, and its presence is significantly correlated with the severity of cognitive symptoms, surpassing the impact of amyloid plaque accumulation [3,4,5,6,48]. Aβ plaques build up in the temporal neocortex in the early stages of AD. This accumulation is associated with disrupted neuronal pathways that connect the prefrontal cortex to other parts of the brain [3,8,50]. Theta oscillations are essential for facilitating communication between the prefrontal cortex and the hippocampus. The activation of the default mode network (DMN) exhibits a negative correlation with theta oscillations, while a significant amyloid burden is associated with reduced DMN activation, subsequently leading to a hyperactivation of the midfrontal theta band [38]. This may reflect a decreased alpha and beta activity and increased theta and delta activity, which are characteristics of AD [14,24,25,26]. According to Schmidt et al. (2013), those with early-to-moderate stages of AD had a reduced alpha/theta ratio, indicating a clear pattern of increased theta and decreased alpha activity in AD patients [50]. Prior studies have demonstrated that, in comparison to cognitively normal subjects, individuals with AD exhibit a higher theta power and lower beta power [51]. Thus, higher levels of TAR and TBR could be indicators of AD.

Many studies have investigated the link between changes in qEEG power spectral patterns in AD and CSF biomarkers like amyloid and tau levels. Smailovic et al. (2018) investigated 637 participants and discovered that diminished CSF amyloid β42 levels correlated with elevated theta and delta power, whereas increased tau levels were associated with reduced alpha and beta power [52]. Del Percio et al. (2025) also discovered that people with ADMCI demonstrated a reduced posterior rEEG alpha activity, which was inversely linked with CSF Aβ levels [53]. These results indicate that Aβ buildup, especially in the initial stages of AD, significantly influences EEG activity, underscoring its effect on brain oscillations throughout the prodromal phases of the condition.

Our findings support those of Bae et al. (2023), who found no significant correlation between TAR and amyloid burden in EC rEEG [37]. However, we found a significant inverse connection between TBR and plasma MDS-OAβ after controlling for age, education, and cognitive performance. In both EO and EC conditions, the frontal, central, parietal, occipital, and temporal lobes had lower TBR values (Table 5 and Table 6). The central and temporal lobes showed the strongest correlations. Unlike Bae et al. [37], who found positive correlations between TBR and amyloid levels in the EC-alone adjusted model, this trend is likely due to cognitive reserve (CR) and methodological variations. Kremen et al. (2022) define cognitive reserve as the brain’s capacity to make up for damage through neuronal efficiency and different cognitive methods [54]. Notably, no significant associations were observed for TAR, suggesting that alterations in this frequency band may be masked by CR, which acts as a buffer against the functional impact of early amyloid deposition on higher-frequency activity.

Our results suggest that the inverse association between TBR and plasma MDS-OAβ levels is moderated by CR, as reflected by educational levels. After balancing the number of participants across education groups to ensure comparability, we observed that the strongest negative associations were consistently present in individuals with ≤6 years of education. In the temporal lobe during the EO condition, the adjusted model showed β = −0.19, SE = 0.06, *p* = 0.003 (Table 6a); and, in the central lobe during the EC condition, β = −0.13, SE = 0.06, *p* = 0.05 (Table 6b). In contrast, participants with 7–12 years or >12 years of education generally exhibited weaker or nonsignificant results (Table 6a,b). Importantly, education was not included as a covariate in the regression models, as stratification inherently addressed group differences. Although education was examined through stratified subgroup comparisons to explore the potential moderating effect of CR, formal interaction testing between MDS-OAβ levels and education was not conducted. Therefore, these findings should be interpreted as exploratory and descriptive rather than inferential evidence of moderation. These findings support the CR hypothesis, which asserts that higher educational levels foster a greater neuronal efficiency and facilitate compensatory cognitive strategies that mitigate the effects of neuropathology [54,55].

This could lessen the effects of amyloid-related neural alterations in the early stages of AD. Collectively, these results suggest that TBR, especially in low-frequency bands, may be a more accurate and sensitive qEEG indicator of amyloid burden than TAR, providing a strong representation of amyloid-related brain changes in older adults, in line with earlier research on DMN hypoactivation [38].

Cholinergic deficiencies in AD may be the cause of the association between Aβ and slower EEG activity. Cholinergic pathways are among the several signaling systems that AD first interferes with. Previous studies have shown that amyloid peptides can impair muscarinic cholinergic receptor-mediated signaling and may inhibit acetylcholine synthesis and release [56]. Acetylcholine synthesis and release are inhibited by Aβ, which also messes with muscarinic receptor coupling. Slow EEG activity has been shown to increase when cortical cholinergic activity is reduced [57]. Slow-frequency activity is increased as a result of Aβ’s negative effects on cholinergic transmission [57,58]. The rapid decrease in EEG rhythms in AD patients may be explained by brain atrophy, as alpha and beta waves are mostly produced in the cortex and propagate through cortical connections [58]. Our results suggest that amyloid oligomerization may be associated with a reduced TBR and altered alpha activity, potentially reflecting early cholinergic dysfunction and neuronal loss. Specifically, the decrease in TBR in the low-frequency bands may indicate that amyloid accumulation disrupts the normal balance of theta and beta oscillations, leading to slowed cortical activity and impaired neural network function.

This research possesses several limitations. First, it is unable to conclude the causal relationship between plasma MDS-OAβ levels and EEG measurements due to the cross-sectional and observational methodology. Second, amyloid load was only measured using plasma MDS-OAβ in this investigation, even though amyloid PET imaging and cerebrospinal fluid Aβ42 (CSF Aβ42) are recognized indicators of brain amyloid pathology. Although MDS-OAβ has not been completely validated as a diagnostic marker for AD, its significant neurotoxicity and established correlations with CSF Aβ42 and amyloid PET suggest its use as a surrogate measure of amyloid accumulation. Third, to ascertain whether TBR can function as a predictive indicator of amyloid buildup and cognitive deterioration, longitudinal research is necessary. Fourth, standardized recording procedures were used to minimize the variability in alertness and attention. However, subtle fluctuations in vigilance between EO and EC conditions cannot be completely excluded. Despite the use of standardized pre-processing procedures to control artifact-related variability, as well as residual ocular or muscular artifacts, these may still have influenced the EEG signals. However, a consistent application of ICA correction and post-processing quality control across all participants likely minimized this bias. Fifth, although the regression models were adjusted for age, education, and cognitive performance, other potential confounders, such as sleep disturbances, depressive symptoms, and cerebrovascular risk factors, were not included as covariates in the statistical models. However, these factors were clinically evaluated during neurological screening, and participants with significant abnormalities were excluded from the study. Therefore, while minor residual confounding cannot be fully ruled out, systematic bias from these variables is unlikely. Lastly, the study cohort was selected from a single neurology department, which could restrict the generalizability of our findings and introduce selection bias. Larger, population-based cohorts should be used in future studies to confirm these correlations and lower uncertainty.

## 5. Conclusions

Our study demonstrates that elevated plasma MDS-OAβ levels are significantly associated with a reduced TBR in older adults, particularly in the parietal and central lobes, across both EO and EC rEEG. No significant associations were observed with TAR. These findings suggest that a decreased TBR may serve as a sensitive non-invasive qEEG marker of early amyloid-related neural alterations, reflecting potential cholinergic dysfunction and subtle neuronal loss. Integrating TBR with plasma MDS-OAβ measurements could provide a practical approach for the early detection and monitoring of AD, though longitudinal studies are warranted to validate its predictive utility.

## Figures and Tables

**Table 1:** Demographics of the study population.

Characteristic	Total (*n* = 174)	MDS Low (<0.78 ng/mL) (*n* = 92)	MDS High (≥0.78 ng/mL) (*n* = 82)
Age group (years) *n* (%)			
<60	13 (7.47)	10 (10.87)	3 (3.66)
60–64	8 (4.60)	4 (4.35)	4 (4.88)
65–69	23 (13.22)	14 (15.22)	9 (10.98)
70–74	27 (15.52)	14 (15.22)	13 (15.85)
75–79	46 (26.44)	25 (27.17)	21 (25.61)
80–84	39 (22.41)	16 (17.39)	23 (28.05)
≥85	18 (10.34)	9 (9.78)	9 (10.98)
Sex—*n* (%)			
Female	123 (70.69)	65 (70.65)	58 (70.73)
Male	51 (29.31)	27 (29.35)	24 (29.27)
Education level—*n* (%)			
Below elementary	42 (24.14)	25 (27.17)	17 (20.73)
Middle school	47 (27.01)	25 (27.17)	22 (26.83)
High school	27 (15.52)	12 (13.04)	15 (18.29)
Over college	58 (33.33)	30 (32.61)	28 (34.15)
Continuous variables (Mean ± SD)			
Age, years	74.87 ± 8.52	73.79 ± 9.01	76.07 ± 7.82
Education, years	10.89 ± 4.44	10.74 ± 4.49	11.04 ± 4.41

Note: Values are presented as n (%) for categorical variables and means ± SD for continuous variables. Abbreviations: *n*, number; %, percentage; SD, standard deviation; MDS, Multimer Detection System–Oligomeric Amyloid-Beta; ng/mL, nano gram per milliliter.

**Table 2:** Comparison of neuropsychological test scores between MDS-OAβ groups.

Neuropsychological Test	Low-MDS Mean (SD)	High-MDS Mean (SD)	*p*-Value
K-Mini-Mental State Examination	23.75 (4.54)	23.02 (5.11)	0.32
Rey Complex Figure Test Copy	27.91 (10.26)	26.44 (10.91)	0.36
Seoul Verbal Learning Test Recall	13.99 (5.83)	13.15 (5.14)	0.31
Digit Span Forward	7.27 (1.47)	7.04 (1.46)	0.29
Digit Span Backward	3.86 (1.75)	3.50 (1.29)	0.12
K-Boston Naming Test	9.50 (3.18)	9.04 (3.22)	0.34

Notes: No significant differences were observed in neuropsychological test scores between high- and low-MDS-OAβ groups. However, these tests remain essential for EEG-related research [25]. Abbreviations: MDS, Multimer Detection System–Oligomeric Amyloid-Beta; SD, standard deviation; *p*-value, probability value; K, Korean; EEG, electroencephalography.

**Table 3:** Mean (SD) of EEG power ratios by lobar region between low- and high-MDS-OAβ groups.

	Lobe	Total Mean (SD)	Low-MDS Mean (SD)	High-MDS Mean (SD)	*p*-Value
TAR	Central	0.04 (0.23)	0.03 (0.24)	0.04 (0.22)	0.86
	Frontal	0.21 (0.20)	0.20 (0.20)	0.21 (0.19)	0.91
	Occipital	−0.15 (0.33)	−0.16 (0.35)	−0.13 (0.30)	0.58
	Parietal	0.01 (0.25)	1.00 × 10^−3^ (0.27)	0.03 (0.24)	0.56
	Temporal	0.04 (0.24)	0.04 (0.26)	0.04 (0.21)	0.96
TBR	Central	0.55 (0.30)	0.57 (0.29)	0.53 (0.32)	0.39
	Frontal	0.77 (0.26)	0.78 (0.25)	0.75 (0.26)	0.41
	Occipital	0.70 (0.35)	0.72 (0.36)	0.66 (0.33)	0.25
	Parietal	0.59 (0.33)	0.62 (0.33)	0.57 (0.33)	0.31
	Temporal	0.74 (0.31)	0.77 (0.31)	0.71 (0.32)	0.22

Abbreviations: TAR, theta–alpha ratio; TBR, theta–beta ratio; MDS, Multimer Detection System–Oligomeric Amyloid-Beta; SD, standard deviation; *p*-value, probability value.

**Table 4:** Correlation coefficients (r) between log-transformed EEG power ratios and MDS-OAβ levels by lobe and condition.

	Lobar Region	r (EO)	*p* (EO)	r (EC)	*p* (EC)
TAR	Frontal	−0.01	0.39	−0.01	0.59
	Central	−0.01	0.39	−0.02	0.25
	Parietal	−2.00 × 10^−3^	0.93	−9.00 × 10^−3^	0.65
	Occipital	0.01	0.72	−0.014	0.66
	Temporal	−0.02	0.35	−0.027	0.14
TBR	Frontal	−0.04	0.02 *	−0.03	0.11
	Central	−0.06	0.02 *	−0.06	0.01 *
	Parietal	−0.053	0.04 *	−0.07	9.00 × 10^−3^
	Occipital	−0.06	0.08	−0.06	0.07
	Temporal	−0.06	0.01 *	−0.05	0.03 *

Note: EEG power ratios were log-transformed. An asterisk denotes statistically significant correlations (*p* < 0.05). Abbreviations: EO, eyes-open; EC, eyes-closed; MDS, Multimer Detection System–Oligomeric Amyloid-Beta; r, correlation coefficients; *p*, probability.

**Table 5:** Lobe association between EEG power ratios and plasma MDS-OAβ levels.

	Lobar Region	Unadjusted Model	Adjusted Model
(a) EO			
TAR	Frontal	β = −0.01 (SE = 0.01), *p* = 0.39	β = −0.01 (SE = 0.01), *p* = 0.43
	Central	β = −0.02 (SE = 0.02), *p* = 0.39	β = −0.01 (SE = 0.02), *p* = 0.44
	Parietal	β = −2.00 × 10^−3^ (SE = 0.02), *p* = 0.927	β = −1.00 × 10^−3^ (SE = 0.02), *p* = 0.97
	Occipital	β = 0.01 (SE = 0.03), *p* = 0.72	β = 0.01 (SE = 0.03), *p* = 0.67
	Temporal	β = −0.02 (SE = 0.02), *p* = 0.35	β = −0.01 (SE = 0.02), *p* = 0.40
TBR	Frontal	β = −0.04 (SE = 0.02), *p* = 0.02 *	β = −0.04 (SE = 0.02), *p* = 0.03 *
	Central	β = −0.06 (SE = 0.02), *p* = 0.03 *	β = −0.05 (SE = 0.02), *p* = 0.03 *
	Parietal	β = −0.05 (SE = 0.03), *p* = 0.05 *	β = −0.05 (SE = 0.03), *p* = 0.06
	Occipital	β = −0.06 (SE = 0.03), *p* = 0.08	β = −0.05 (SE = 0.03), *p* = 0.09
	Temporal	β = −0.06 (SE = 0.02), *p* = 0.01 *	β = −0.05 (SE = 0.02), *p* = 0.02 *
(b) EC			
TAR	Frontal	β = −8.00 × 10^−3^ (SE = 0.01), *p* = 0.59	β = −7.00 × 10^−3^ (SE = 0.01), *p* = 0.64
	Central	β = −0.02 (SE = 0.02), *p* = 0.25	β = −0.02 (SE = 0.02), *p* = 0.25
	Parietal	β = −9.00 × 10^−3^ (SE = 0.02), *p* = 0.65	β = −0.01 (SE = 0.02), *p* = 0.64
	Occipital	β = −0.01 (SE = 0.03), *p* = 0.66	β = −0.02 (SE = 0.03), *p* = 0.55
	Temporal	β = −0.03 (SE = 0.02), *p* = 0.14	β = −0.03 (SE = 0.02), *p* = 0.11
TBR	Frontal	β = −0.03 (SE = 0.02), *p* = 0.11	β = −0.03 (SE = 0.02), *p* = 0.14
	Central	β = −0.06 (SE = 0.03), *p* = 0.01 *	β = −0.06 (SE = 0.02), *p* = 0.02 *
	Parietal	β = −0.07 (SE = 0.03), *p* = 0.01 *	β = −0.07 (SE = 0.03), *p* = 6.00 × 10^−3^ *
	Occipital	β = −0.06 (SE = 0.03), *p* = 0.07	β = −0.07 (SE = 0.03), *p* = 0.04 *
	Temporal	β = −0.05 (SE = 0.02), *p* = 0.03 *	β = −0.05 (SE = 0.02), *p* = 0.02 *

Notes: EEG power ratios were log-transformed. Unadjusted models are without covariates; adjusted models control for age, education level, and K-MMSE. An asterisk (*) denotes statistically significant correlations (*p* ≤ 0.05). Abbreviations: MDS-OAβ: Multimer Detection System–Oligomeric Amyloid-β; K-MMSE, Korean Mini-Mental State Examination; TAR, theta–alpha ratio; TBR, theta–beta ratio; β = standardized regression coefficient; SE = standard error; *p* = probability value.

**Table 6:** Lobe association between EO and EC EEG power ratios and plasma MDS-OAβ levels, grouped by education level.

Lobar Region	Education Group	Unadjusted Model	Adjusted Model
(a) EO			
Central	Education ≤ 6	β = −0.18 (SE = 0.07), *p* = 6.1 × 10^−3^ *	β = −0.19 (SE = 0.06), *p* = 3.4 × 10^−3^ *
	Education 7–12	β = 0.03 (SE = 0.06), *p* = 0.63	β = −0.01 (SE = 0.06), *p* = 0.87
	Education > 12	β = −0.07 (SE = 0.06), *p* = 0.23	β = −0.07 (SE = 0.06), *p* = 0.22
Temporal	Education ≤ 6	β = −0.18 (SE = 0.06), *p* = 3.7 × 10^−3^ *	β = −0.19 (SE = 0.06), *p* = 1.8 × 10^−3^ *
	Education 7–12	β = −0.02 (SE = 0.05), *p* = 0.69	β = −0.03 (SE = 0.05), *p* = 0.62
	Education > 12	β = −0.02 (SE = 0.05), *p* = 0.70	β = −0.02 (SE = 0.05), *p* = 0.70
(b) EC			
Central	Education ≤ 6	β = −0.12 (SE = 0.06), *p* = 0.07	β = −0.13 (SE = 0.06), *p* = 0.05 *
	Education 7–12	β = −0.05 (SE = 0.06), *p* = 0.40	β = −0.11 (SE = 0.05), *p* = 0.06
	Education > 12	β = −0.11 (SE = 0.07), *p* = 0.11	β = −0.12 (SE = 0.07), *p* = 0.08
Parietal	Education ≤ 6	β = −0.05 (SE = 0.07), *p* = 0.46	β = −0.08 (SE = 0.07), *p* = 0.27
	Education 7–12	β = 0.01 (SE = 0.08), *p* = 0.92	β = −0.03 (SE = 0.07), *p* = 0.68
	Education > 12	β = −0.06 (SE = 0.07), *p* = 0.40	β = −0.09 (SE = 0.07), *p* = 0.20

Notes: EEG power ratios were log-transformed. Unadjusted models are without covariates; adjusted models control for age and K-MMSE. Education level was used for stratification and not included as a covariate. An asterisk (*) denotes statistically significant correlations (*p* ≤ 0.05). Abbreviations: MDS-OAβ: Multimer Detection System–Oligomeric Amyloid-β; β = standardized regression coefficient; SE = standard error; *p* = probability value.

## Data Availability

The data supporting the findings of this study are available upon reasonable request from the corresponding author, Y.C.Y, via email. Due to confidentiality agreements and institutional policies protecting sensitive patient information from the hospital where the study was conducted, the data cannot be made publicly available. Requests will be evaluated to ensure compliance with ethical and legal standards.

## Supplementary Materials

The following supporting information can be downloaded at https://www.mdpi.com/article/10.3390/jcm14228069/s1. Figure S1: 19 Scalp electrodes positioned based on the international 10–20 system.
