# Supplementary figures and images for: Association of qEEG TAR and TBR During Eyes-Open and Eyes-Closed with Plasma Oligomeric Amyloid-β Levels in an Aging Population
Source: J Clin Med. 2025 Nov 14;14(22):8069. doi: 10.3390/jcm14228069 (PMC12653316; doi:10.3390/jcm14228069)

**Supplementary Figure S1.** 19 Scalp Electrodes Positioned based on the International 10-20 System.

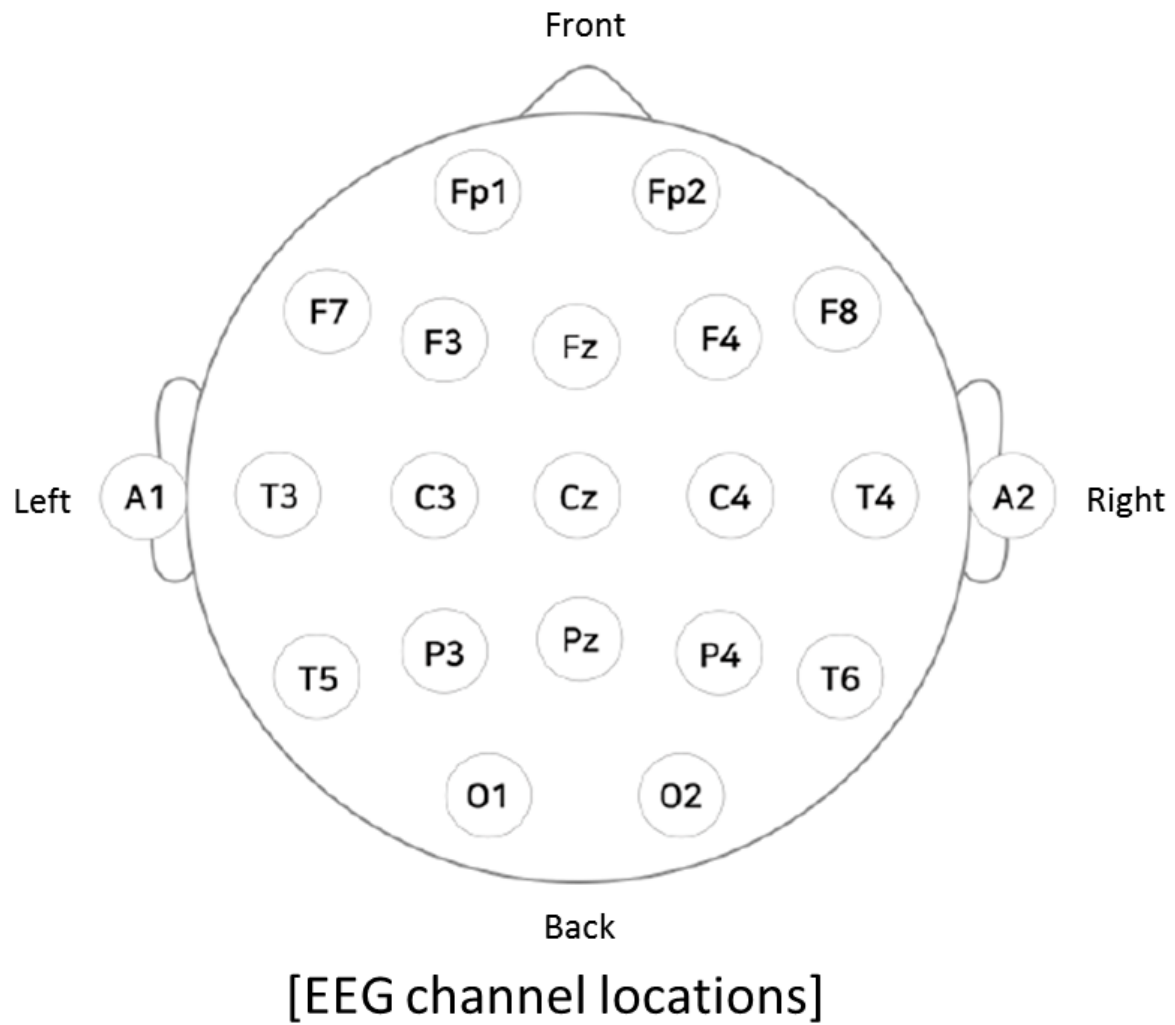

Supplement: Supplementary file 1 [file jcm-14-08069-s001.zip › jcm-3936922-supplementary.pdf]
